# FACTORS LEADING TO DISCONTINUOUS USE OF GOVERNMENT-SPONSORED LONG-TERM CARE 2.0 SERVICE PLAN IN TAIWAN

**DOI:** 10.1093/geroni/igae098.3191

**Published:** 2024-12-31

**Authors:** Ya-Mei Chen, Shih-Cyuan Wu

**Affiliations:** National Taiwan University, Taipei City, Taipei, Taiwan (Republic of China); National Taiwan University, Taipei, Taipei, Taiwan (Republic of China)

## Abstract

The Long-Term Care 2.0 (LTC 2.0) Plan, initiated in Taiwan in 2017, respectively, aimed to provide home- and community-based services (HCBS) for individuals in need. However, a significant proportion discontinued usage within six months, prompting an investigation into influencing factors. This study, analyzing LTC 2.0 data from 2018 to 2021 for older adults in two cities, tracked 59,151 service users for up to a year after their initial assessments through three application stages (assessment to service prescription, service utilization, and continuous service utilization for more than six months). Key factors affecting discontinuation included age, middle-low income family status, behavioral problems, informal caregivers, and foreign caregivers. Individuals with informal caregivers had a higher likelihood of obtaining service prescriptions (OR=1.82***) and utilizing LTC services (OR=1.33***), but were prone to discontinuing LTC 2.0 during continuous service utilization (OR=0.78***). Those with foreign caregivers were less likely to progress beyond the service utilization stage (OR=0.77***), but were inclined to maintain usage once started (OR=1.16***). Similarly, those aged over 85 and individuals with behavioral problems were less likely to progress beyond utilization but inclined to maintain usage (Age OR=1.22***; Behavioral problems OR= 1.14***). In contrast, individuals from middle-low income families were more likely to be prescribed and initiate usage but less likely to maintain it (OR= 0.77***). The presence of informal/foreign caregivers significantly influenced utilization status across these three stages, emphasizing the need for services development that address not only users’ needs but also the needs of both formal and informal caregivers.

